# Cancer Associated Fibroblasts and Tumor Growth: Focus on Multiple Myeloma

**DOI:** 10.3390/cancers6031363

**Published:** 2014-06-27

**Authors:** Kim De Veirman, Luigia Rao, Elke De Bruyne, Eline Menu, Els Van Valckenborgh, Ivan Van Riet, Maria Antonia Frassanito, Lucia Di Marzo, Angelo Vacca, Karin Vanderkerken

**Affiliations:** 1 Department of Hematology and Immunology, Myeloma Center Brussels, Vrije Universiteit Brussel (VUB), Brussels 1090, Belgium; E-Mails: luigiarao@gmail.com (L.R.); elke.de.bruyne@vub.ac.be (E.D.B.); eline.menu@vub.ac.be (E.M.); els.van.valckenborgh@vub.ac.be (E.V.V.); ivan.vanriet@uzbrussel.be (I.V.R.); 2 Department of Biomedical Sciences and Human Oncology, Section of Internal Medicine, University of Bari Medical School, Bari I-70124, Italy; E-Mail: luciana1976@libero.it (L.M.); angelo.vacca@uniba.it (A.V.); 3 Stem Cell Laboratory, Division of Clinical Hematology, Universitair Ziekenhuis Brussel (UZ Brussel), Brussels 1090, Belgium; 4 Department of Biomedical Sciences and Human Oncology, Section of General Pathology, University of Bari Medical School, Bari I-70124, Italy; E-Mail: antofrassanito@gmail.com

**Keywords:** multiple myeloma, hypoxia, cancer associated fibroblasts

## Abstract

Cancer associated fibroblasts (CAFs) comprise a heterogeneous population that resides within the tumor microenvironment. They actively participate in tumor growth and metastasis by production of cytokines and chemokines, and the release of pro-inflammatory and pro-angiogenic factors, creating a more supportive microenvironment. The aim of the current review is to summarize the origin and characteristics of CAFs, and to describe the role of CAFs in tumor progression and metastasis. Furthermore, we focus on the presence of CAFs in hypoxic conditions in relation to multiple myeloma disease.

## 1. Introduction

It is now well-accepted that growth and progression of a multitude of malignancies depends on interactions of the tumor cells with their local tumor microenvironment. Distinct cell types of this microenvironment including endothelial cells, immune cells and fibroblasts contribute to tumorigenesis by secretion of cytokines and/or direct cell-cell contact [1]. A particular subpopulation of fibroblasts, the so-called cancer associated fibroblasts (CAFs), has recently raised the interest of many researchers due to their active participation in tumor growth and invasion. Despite the fact that they are the most prominent cell type in the tumor microenvironment of many cancers, the “CAF population” remains poorly defined due to a lack of CAF-specific markers and a high heterogeneity [2,3,4].

Normal fibroblasts maintain tissue homeostasis, regulate inflammation and epithelial differentiation, and play a crucial role in the wound healing process. They exert distinct functions including the secretion of extracellular matrix (ECM) components and ECM-degrading enzymes like matrix metalloproteinases. During wound healing, they recruit endothelial precursor cells and promote angiogenesis. In addition, they also promote epithelial cell growth and induce epithelial-mesenchymal transition to bridge wounds. Under the influence of transforming growth factor beta (TGF-β), normal fibroblasts become myofibroblasts which are responsible for wound contraction. In contrast to normal fibroblasts and myofibroblasts, CAFs are continuously activated and are not able to revert into a normal phenotype or undergo apoptosis. They are defined as activated fibroblasts which are present in the stroma surrounding the tumor [5,6].

## 2. Characterization of CAFs

Fibroblasts form a heterogeneous cellular component of connective tissues. Tissue damage induces their activation and increased collagen, cytokine and growth factor secretion, which regulate the wound healing processes. CAFs are a subpopulation of fibroblasts found in the tumor microenvironment [7]. Despite the increased number of CAF markers, no unique marker could be identified to distinguish CAFs from normal fibroblasts, or from other cells present in the tumor microenvironment [4]. CAFs are mostly characterized based on three important features: (1) expression of fibroblast markers vimentin, fibroblast specific protein 1 (FSP1) and fibroblast activating protein (FAP); (2) expression of activation marker αSma and aggressive/invasive markers including Thy1, thrombospondin-1 (Tsp-1), plateled derived growth factor receptor (PDGFR α/β), tenascin-C and matrix metalloproteinase 3 (MMP-3); and (3) increased cytokine and growth factor expression including vascular endothelial growth factor (VEGF), transforming growth factor-beta (TGF-β), hepatocyte growth factor (HGF), epidermal growth factor (EGF) and fibroblast growth factor-2 (FGF-2) (Table 1) [8,9,10,11,12,13,14,15,16,17,18,19,20,21,22].

**Table 1:** Summary of the markers for cancer associated fibroblasts.

Fibroblast markers	Ref.	Activation (aggressive) markers	Ref.
Fibroblast specific protein 1 (FSP-1)	[10,15]	Alpha smooth muscle actin (αSma)	[23]
Vimentin	[10,16]	Podoplanin	[14]
Fibroblast activating protein (FAP)	[9,17]	Thrombospondin (Tsp-1)	[10]
		Thy1 (CD90)	[13]
		Tenascin C (TN-C)	[10]
		Matrix metalloproteinase (MMP)	[19]
		Neuron glial antigen 2 (NG2)	[12]
		Periostin	[20]
		Palladin	[8]
		PDGFR (α/β)	[12,21,22]

## 3. Origin and Generation of CAFs

It has been described that CAFs originate from at least four different cellular components. Normal fibroblasts or fibroblast precursors are the most obvious cell type, which can differentiate into CAFs upon stimulation with paracrine signals. TGF-β has been recognized as the most potent inducer of transformation of fibroblasts to CAFs [5]. Recently, a cell adhesion molecule called extracellular matrix metalloproteinase inducer (EMMPRIN) has been described as a CAF activator, and is overexpressed by various types of cancers [24]. In ovarian cancer, distinct microRNAs (e.g., miR214, miR31 and miR155) that reprogrammed the normal fibroblasts into CAFs were also identified [25].

It is widely assumed that CAFs may also develop from mesenchymal stem cells (MSCs). Both cell types show more similarities than differences including the expression of cell surface markers (HLA-DR, CD29, CD90, CD44, CD73, CD106 and CD117) and cytoskeleton proteins like vimentin and αSma. Although the relationship between both cell types has not established, fibroblasts are considered as more differentiated cells with a more restricted differentiation potential. Importantly, CAFs showed a higher proliferation rate and increased production of cytokines, chemokines and growth factors (including VEGF, TGF-β, TNF-α and interleukins) when compared to MSCs [26]. Spaeth and colleagues showed that conditioned medium from ovarian carcinoma cells “Skov3” can activate MSCs, leading to increased expression of CAF-like surface markers [10]. Furthermore, Direkze and colleagues transplanted green fluorescent protein (GFP) αSma+ MSCs from a male donor into a female recipient and found 25% positivity for GFP in the total myofibroblast population, indicating that MSCs may represent a potential source for CAFs [25]. Mi and colleagues identified osteopontin as a possible regulator of MSCs transformation into CAFs [27]. Endothelial-mesenchymal transition (EndMT) and epithelial-mesenchymal transition (EMT) are also recognized as a source of CAFs [5,28]. Zeisberg and colleagues demonstrated that EndMT, which is partially regulated by TGF-β1 signaling, contributes to CAF formation in a melanoma and pancreatic carcinoma model [29].

## 4. Mechanism of CAFs Activation

CAFs isolated from different types of human carcinomas including breast, lung, prostate and ovarian actively take part in tumor development and growth. Under influence of tumor cells CAFs show morphological differences and increased secretory and metabolic activities compared to normal fibroblasts [30]. As Gabbiani and colleagues described in their papers, CAFs are fibroblastic cells, characterized by a big indented nucleus, an extensive arrangement of actin microfilaments, enlarged endoplasmic reticuli and Golgi complex. This activated ultra-structural state reflects enhanced secretion of enzymes (metalloproteinases, their inhibitors and other proteinases), growth factors (TGF-β, CXCL12, PDGF-AA, IL-6, FGF-2, EGF family members, IGF-1 and HGF) and matrix proteins (collagen type I, laminin, fibronectin, ED-A) [31].

A recent report by Peng and colleagues reveals a list of up-regulated genes in CAFs isolated from primary breast infiltrating ductal carcinomas of grade II compared to the normal fibroblasts. It is shown that CAFs over-express genes involved in cell proliferation (CENPF, PLK1, BUB1, KIF15, CRIP1) and growth factors (FGF-2, VEGFC, PDGFC, CASC5, HGFAC). *In vitro* experiments furthermore showed that co-culturing of the MDA-MB-231 breast cancer cell line with human CAFs isolated from mammary tissue resulted in an increased proliferation rate with a great proportion of tumor cells in S phase compared to tumor cells co-cultured with normal fibroblasts [32]. CAFs morphological and secretory features (growth factors, chemokines and cytokines) are necessary to modify the surrounding microenvironment and to communicate with tumor cells and other stromal cells [8,33].

In different tumor contexts soluble factors secreted by CAFs act in a paracrine and/or autocrine manner to sustain tumor cell proliferation and maintain the activated CAF phenotype [34,35]. Notwithstanding the great complexity of the soluble factors milieu, the most important growth factors secreted by CAFs are TGF-β and CXCL12. The importance of the TGF-β and CXCL12 loops is well explained in breast cancer [36,37]. Kojima and colleagues demonstrated that the acquirement of an activated CAF phenotype by mammary fibroblasts and the associated tumor stimulating properties are related to the activation of TGF-β and CXCL12 autocrine loops [38]. 

Up till now TGF-β is considered as one of the major mediators of CAFs activation. TGF-β, a pleiotropic cytokine secreted by different cell types, including epithelial cells and fibroblasts, is stored as a reservoir in the ECM [39]. TGF-β1 binds its receptor TGFβRII on fibroblast membranes, and forms a heterodimeric activated-receptor complex recruiting TGFβRI (ALK5). This complex catalyzes the activation of the SMAD dependent or independent signaling cascades. The first pathway included the phosphorylation of RSMADs (SMAD 1, 2, 3, 5 and 8) and the binding of SMAD4. As a result, the complex moves to the nucleus where it acts as a transcription factor of genes encoding for growth factors (PDGF, VEGF), matrix metalloproteinases (MMP-9 and TIMP), and genes involved in CAFs differentiation and activation (αSma) and regulators of the EndMT process (SNAIL, ZEB1/2). On the other hand, TGF-β could also trigger the activation of SMAD independent signaling cascades including ShcA, RAC/CDC42, RAS, TRAF6, TAK1, PI3K, MAP3K1 and RhoA pathways. Recently Webber and colleagues reported a new mechanism of inter-cellular communication between tumor cells and fibroblasts mediated by exosomes expressing TGF-β that result in CAFs activation [40].Interestingly Qiong Li and colleagues demonstrated that TGF-β mediated CAFs activation could be regulated by SMAD7 and miR21. SMAD7 physically binds to SMAD2 and SMAD3 which in turn blocks the TGF-βR-dependent signaling [39]. It is shown in fibroblasts that TGF-β stimulates miR21 maturation that acts by decreasing SMAD7 protein expression through translation inhibition. In this way the SMAD2/SMAD3 complex triggers the signaling cascade and induces CAFs activation [41]. Interestingly, in colitis associated-cancers, epiregulin (EREG) was found to be an important factor secreted by CAFs sustaining activated phenotype through an autocrine loop. EREG secreted by CAFs also acts in a paracrine way on epithelial cells increasing their proliferation rate through the activation of the ERK pathway [42].

Several studies on breast, pancreatic, prostatic and ovarian cancers report that treatment of normal fibroblasts with tumor cell conditioned media triggers phenotypic modifications such as expression of activation markers and increased CAFs secretory properties. Solid tumor cells can secrete growth factors, such as endothelin-1, thrombin, PDGF, FGF-2 and CXCL12, that can stimulate directly or indirectly the myofibroblastic differentiation [43,44]. PDGF, an important factor in the wound healing process, is not able to directly stimulate fibroblast activation. However, it increases the TGF-β release by other stromal cells (e.g., macrophages) and this can induce fibroblast activation indirectly. TGF-β stimulation will also induce PDGF receptor expression in the fibroblasts and therefore PDGF is mainly considered to mobilize the stromal cells called “stromalization” [45]. Recently, Brentnall and colleagues demonstrated that exogenous introduction of palladin into normal human dermal fibroblasts induces them to become myofibroblasts. It is reported that the activated myofibroblasts do not only show differences in morphology but also in phenotypic and proteomic profiles [8]. 

Besides effects mediated by growth factors, CAFs activation processes depend on many other stimuli that modify the homeostatic microenvironment. Tumor cells’ aberrant proliferation disrupts normal ECM composition, causing the delivery of these diffusible soluble factors as well as mechanical stress. Solid tumors are mostly characterized by matrix stiffness modifications that are involved in the CAFs activation and function. Fibroblasts perceive mechanical signals from the ECM through integrin receptors that physically link the ECM to the cytoskeleton acting as force transducers. In fibroblastic cells it is reported that RhoA, a small guanosine 5'-triphosphatase, is an important link in integrin-dependent mechanotransduction [46]. RhoGAP is a protein involved in cytoskeleton contraction as a consequence of mechanical stress transmission. Recently Gillette and colleagues used a TetO-p190B/MMTV-rtTA murine model, characterized by mammary gland epithelial cells over-expressing p190B RhoGAP. In this model ECM associated with hyperbranched terminal end buds (TEB) appears thick and collagen enriched. Epithelial cells over-expressing p190B secrete large amounts of TGF-β and act in a paracrine manner on surrounding fibroblasts. Activated fibroblasts increase the ECM deposition of collagen (Col1a1, Col3a1, Col6a1), laminin-α1 and fibronectin as main extracellular components. Alteration of the ECM composition creates mechanical tension that in turn stimulates CAFs activation [47].

**Figure 1:**
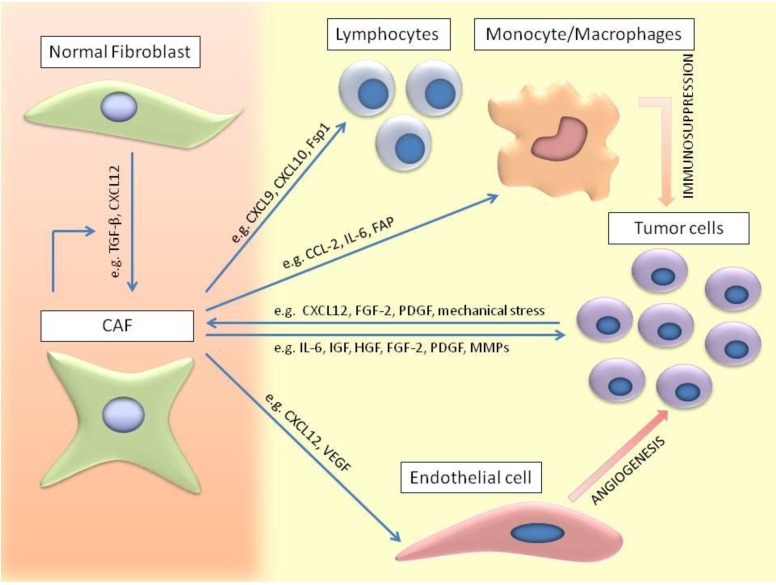
Schematic overview of CAFs involvement in tumor growth and progression. Local fibroblasts, generally considered as the major source of CAFs, can be stimulated by autocrine/paracrine TGF-β and CXCL12. CAFs are able to induce tumor progression by direct and indirect effects. They secrete growth factors (e.g., IL-6, IGF, HGF, FGF-2, PDGF) and enzymes like matrix metalloproteinases (MMPs) which are able to induce tumorprogression and metastasis. Importantly, they modify the surrounding tumor microenvironment by actions on endothelial cells and immune cells. Secretion of pro-angiogenic factors CXC12, VEGF, FGF, IL8/CXCL8 and PDGF-C by CAFs recruit endothelial cells and their precursors in order to stimulate tumor angiogenesis. Furthermore, CCL-2, IL-6, FAP, IL-4, hyaluronan and IL-8 secretion by CAFs played a role in monocyte/macrophage recruitment, differentiation and M2 polarization resulting in immunosuppression. CXCL9, CXCL10, CXCL12 and Fsp1 were mentioned to promote lymphocyte recruitment and induce immunosuppression by an increase in Th2, Th17 and regulatory T-cells. All these effects on neighboring cells lead to increased tumor cell proliferation. In turn, tumor cells secrete growth factors and induce mechanical stress causing CAFs activation.

All these data confirm that factors secreted by tumor cells and changes in matrix composition and organization synergistically collaborate in stimulating fibroblast activation, as shown in Figure 1.

Besides the influence of tumor cells on CAFs activation, it is important to realize that cytotoxic therapy can also induce fibroblast activation. Ravani *et al.* [22] showed that radiation could induce changes in the stromal microenvironment accompanied by neoplastic progression *in vivo*. Furthermore, Lotti and colleagues [48] demonstrated that chemotherapy-treated CAFs enhanced cancer stem cell self-renewal and *in vivo* tumor growth. 

## 5. CAFs Involvement in Tumor Development

CAFs involvement in tumor progression has been demonstrated independently in several laboratories using murine xenograft models. Co-injection of CAFs with tumor cells resulted in enhanced tumor formation. In contrast, co-injection of normal fibroblasts with tumor cells was not able to give the same results in terms of tumor take, growth rate and tumor load [49,50]. For example, in prostatic carcinoma, Olumi and colleagues [51] demonstrated that immortalized prostatic epithelial cells give rise to large tumors after co-injection with prostatic CAFs. On the contrary no tumor formation is observed after injection of immortalized prostatic epithelial cells alone or in combination with normal human fibroblasts. These enlarged tumors were closely associated with increased tumor cell proliferation and angiogenesis suggesting an important role of CAFs in sustaining direct and indirect tumor growth [51]. It is widely accepted that in epithelial tumors, CAFs are the most prevalent cellular stromal component, providing signals to promote carcinogenesis and to support tumor development and metastasis [28]. Clinical data report that the percentage of CAFs in solid carcinomas such as prostatic, pancreatic, ovarian and breast cancer is correlated with a higher grade of malignancy and poor prognosis. Franco and colleagues combined rat urogenital sinus mesenchyme and Dunning Prostate Adenocarcinoma epithelium with or without different percentages of CAFs to demonstrate that the influence of this cell population on prostatic gland organization and on tumorigenesis is closely correlated to the stromal composition. Interestingly, a small percentage of CAFs (25%) is already able to modify the physiologic epithelial compartment structure [30]. 

CAFs and tumor cells create a cytokine network, working directly on both cell types which results in increased tumor progression. In malignant pleural mesothelioma, Li and colleagues [52] assessed the importance of PDGF-AA, FGF-2 and HGF cytokines networks between tumor cells and CAFs. They observed that tumors, developed after orthotopical inoculation of human MSTO-211 cells (a mesothelioma cell line) in the thoracic cavity of SCID mice, presented great CAFs infiltration. *In vitro* co-culture and migration experiments using several human mesothelioma cell lines (MSTO-211, Y-MESO-14) and human embryonic lung fibroblast cell lines showed that mesothelioma cells secreted FGF-2 and PDGF-AA. These growth factors attract and activate fibroblasts in the tumor region increasing their cell growth and HGF secretion [52]. In gastric cancer, IL-6 secreted by fibroblasts is considered an important factor involved in the early stage of tumorigenesis. Comparison of IL-6 expression on human sections, both from healthy donors and diseased patients, and on sections obtained from a mouse model of chemically induced gastric cancer demonstrated higher IL-6 expression by CAFs. *In vitro* experiments showed that conditioned media of gastric cancer cell lines induced IL-6 secretion by fibroblasts through IL-1 involvement. In turn, IL-6 acts in a paracrine manner increasing tumor gastric cell line proliferation through STAT3 signaling [53].

Moreover, experimental data from the most widespread solid carcinomas demonstrated that CAFs directly modulate tumor growth by secreting factors with oncogenic and mitogenic functions that are able to increase tumor cell proliferation and to protect them from apoptosis. CAFs can also act indirectly by secreting chemotactic factors to recruit other stromal cell types into the tumor compartment and also by secreting pro-angiogenic factors resulting in increased angiogenesis. In fact, CAFs release a great number of pro-angiogenic factors including VEGF, CXCL12, FGF, IL-8/CXCL8 and PDGF-C in the ECM to recruit other stromal cell types such as endothelial cells and their precursors in order to stimulate tumor angiogenesis and vasculogenesis [54]. For example, it is reported that CXCL12 secreted by myofibroblasts has an important role in breast cancer tumorigenesis. Binding of CXCL12 to its cognate receptor CXCR4 stimulates the activation of different intracellular pathways involved in proliferation, migration and invasion of cancer cells. The release of CXCL12 by CAFs is also correlated to the mobilization of the endothelial precursor cells expressing CXCR4 from the bone marrow resulting in *de novo* induction of vasculogenesis [36]. In pancreatic tumors, CXCL12 secreted by CAFs acts on carcinoma cells, thereby stimulating CXCL8 production. CXCL12 and CXCL8 synergistically increase endothelial cell proliferation and migration, boosting the angiogenic pathway [55].

Several reports also demonstrated the importance of the microenvironment in inducing the cancer stem cell (CSC) phenotype. CSC are a small subpopulation of cells that play a crucial role in tumor initiation, progression an refractoriness. Vermeulen *et al.* [56] showed that myofibroblast-secreted factor HGF enhanced Wnt signaling activity in colon cancer cells, which subsequently stimulated the CSC feature. Tsuyada and colleagues found that increased CCL-2 expression by activated fibroblasts was able to stimulate the stem cell specific phenotype in breast cancer cells [57]. 

## 6. CAFs Involvement in Invasion and Metastasis

It is well known that CAFs actively participate to the creation of a niche where cancer cells can proliferate and also sustain their dissemination by setting up the most advantageous “soil” conditions where the “seeds” can grow better. 

Cancer metastasis is a multistep process correlated to the invasive and migratory behavior of tumor cells that escape from the site of origin, enter in the blood circulation and colonize distant organs. This complex series of events can be regulated intrinsically by the tumor cells and also extrinsically by the surrounding stroma. CAFs could also be considered as modulators of local tumor cell invasion and regulators of the tumor cell spread to distant secondary metastatic sites. It is demonstrated that CAFs involvement in tumor cell metastasis could not only be dependent on the release of soluble factors but also on their physical involvement [58,59]. Recently, Gaggioli and colleagues demonstrated that myofibroblasts are able to create tunnels through the matrix, clearing a path that allows the cancer cells to follow behind [60]. This mechanism is related to the CAFs ability of ECM remodeling through the secretion of proteases and metalloproteineases. For example, in pancreatic cancer, CAFs expressing palladin are able to promote *in vitro* and *in vivo* invasion of tumor cells as a consequence of their increased ability to degrade matrix proteins [61]. 

Duda and colleagues [62] demonstrated that metastatic tumor cells formed complexes with their “own soil”, including stromal cells, and travel together to colonize the lungs. The presence of stromal cells increases the viability of the tumor cells in the blood stream circulation, protecting them from apoptosis. In addition, it is reported that after secondary site colonization the co-travelling CAFs confer growth advantage to the tumor cells, favoring the formation of the metastatic colonies [62].

In the last decades, many scientific reports described that the role of CAFs in the invasion process is also related to the secretion of soluble factors that trigger cancer cell migration. One of the most important chemotactic factors released by CAFs is HGF. It binds the cMET receptor on the tumor cells, thereby stimulating tumor invasiveness and growth. Among the several factors with chemotactic properties secreted by CAFs Karnoub and colleagues reported that in breast cancer CCL5 is involved in metastasis formation [63]. In addition, in breast carcinoma mouse models, *in vivo* experiments demonstrated the role of S100A4 expressed by stromal fibroblasts to stimulate metastasis formation. The authors reported that after co-injection of mouse mammary CSML100 tumor cells with S100A4 (+/+) fibroblasts mice developed metastatic tumors in the lung. In contrast, mice lacking S100A4 expression showed inhibition of the metastatic formation [64]. Pena and colleagues identified stanniocalcin 1 (STC1) as a protein secreted by CAFs stimulating the metastatic behavior of colorectal tumor cells. This was demonstrated by an *in vivo* experiment using an orthotopic colon cancer model whereby HCT116 colorectal cancer cells were co-injected respectively with fibroblasts isolated from wild type mice or from *STC1*^−/−^ mice. Tumors that developed from HCT116 cells and *STC1*^−/−^ MEF co-injection were smaller compared to tumors in tcontrol group and interestingly the treated experimental group showed decreased small metastatic lesions [65]. All these data clearly show that CAFs play an active role in metastasis, supporting tumor cell invasion of local tissues or increasing tumor cell viability through the bloodstream. Importantly, not all the tumor cells are able to induce metastatic tumors. The microenvironment could be a promoter or an obstacle to the new tumor formation. It can be assumed that CAFs, as prominent stromal cell component of solid tumors, modify the microenvironment in the secondary setting creating the optimal conditions for secondary tumor development. Obviously, mechanisms by which CAFs support metastasis formation needs to be better elucidated and hopefully this could give rise to new therapeutic strategies in order to avoid the spread of disease.

## 7. Interaction of CAFs with Immune Cells

A recent review by Raz and colleagues described the role of fibroblasts on immune cell recruitment in cancer. Briefly, cytokines and chemokines secreted by CAFs recruit leukocytes, monocytes/macrophages and mast cells to the tumor, thereby contributing to the cancer-related inflammation [66]. CCL2 secretion by CAFs showed a role in monocyte/macrophage recruitment in lymphoma, breast and melanoma tumors, and was correlated with increased tumor progression. Furthermore, secretion of IL-6, FAP, IL-4 and IL-8 by CAFs was mentioned to play a role in macrophage differentiation or M2 polarization resulting in an immunosuppressive microenvironment. Tumor necrosis factor (TNF) and IL-6 were identified as mast cell chemoattractants, which in turn can result in enhanced tumor invasion and metastasis. CXCL9, CXCL10, CXCL12 and FSP1 production by CAFs promote recruitment of T lymphocytes into the tumor; however the number of cytotoxic T cells *versus* tumor suppressive cells is shifted. CAFs induce immunosuppression by an increase in Th2 cells, Th17 cells and regulatory T-cells. Importantly, fibroblasts not only recruit myeloid cells into tumors via cytokine and chemokine production, but also by modifying the ECM [66,67,68]. Kobayashi and colleagues demonstrated that hyaluronan (HA) is essential for recruitment of macrophages and leukocytes, a process that is likely mediated via toll-like receptors (TLRs) [69].

## 8. Role of CAFs in Therapy Resistance

Activated fibroblasts not only promote tumor growth but play a major role in therapy resistance. Müerköster *et al.* [70] demonstrated that human pancreatic carcinoma cell lines became less sensitive to etoposide treatment when co-cultured with fibroblasts. As IL-1β was described as an important regulator of chemoresistance, an IL-1β receptor blockade antibody was able to abolish the drug resistance effects during cocultivation [70]. Another component of the microenvironment linked to chemoresistance is the ECM molecule hyaluronan (HA). Activated fibroblasts showed increased HA expression, an important matrix determinant in the physical barrier to treatment. Enzymatic targeting of stromal HA, combined with the standard chemotherapy, resulted in a better overall survival of pancreatic ductal adenocarcinoma [71]. Furthermore, Crawford and colleagues found that PDGF-C was upregulated in CAFs derived from resistant tumors. As PDGF-C promotes angiogenesis, PDGF-C inhibitors may be combined with VEGF antagonists to overcome resistance to anti-VEGF therapy [72]. 

## 9. CAFs in Multiple Myeloma

Multiple Myeloma (MM) is an incurable hematological malignancy characterized by the abnormal growth of malignant monoclonal plasma cells in the bone marrow. Clinical features of this disease include anemia, bone pain, renal failure, frequent occurrence of infections and hypercalcaemia. The supportive role of the bone marrow microenvironment in MM development has been widely accepted. The cellular compartment includes fibroblasts/bone marrow stromal cells (BMSC), endothelial cells, osteoclasts, osteoblasts and immune cells, while the non-cellular compartment is composed of a liquid milieu containing growth factors, chemokines and cytokines. MM cells home to the bone marrow and adhere to ECM and BMSC. This interaction activates distinct signaling cascades and confers tumor cells resistance. BMSC also release pro-angiogenic factors contributing to the BM neovascularization, an important feature in MM progression. The imbalance between osteoblasts and osteoclasts results in bone resportion [73,74]. Importantly, primary mouse and human MM cells cannot survive *in vitro* without stromal support, indicating the direct dependence of MM cells on the associated stroma [75].

**Figure 2:**
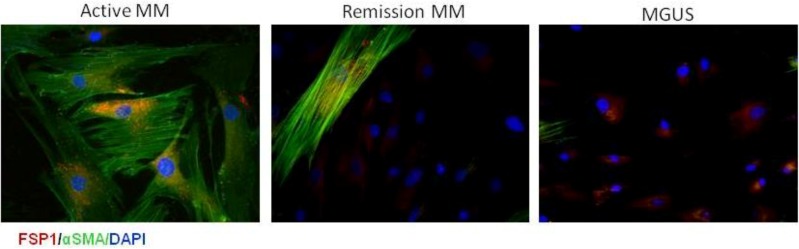
Immunofluorescence staining of purified bone marrow CAFs with αSma (green) and FSP1 (red) in representative MM patients and the benign monoclonal gammopathy (MGUS) patients. Cell nuclei stained with DAPI. Original magnifications 200×.

Until now, only very little data exist about the role of CAFs in hematological malignancies. Among the different cell types that create the bone marrow niche little is known about the role of CAFs in the pathophysiology of MM. Recently, we were the first to describe phenotypic features and functional involvement of CAFs in MM development. CAFs were defined as stromal cells expressing different levels of FSP1, αSma and FAP proteins. Interestingly, a direct correlation was demonstrated between the aggressiveness and progression of disease and the proportion of CAFs in the bone marrow of patients (Figure 2). The results were also confirmed in the murine 5T33MM syngeneic model where an increased CAFs percentage was observed in the bone marrow of the 5T33MM diseased animals compared to naïve mice. As described by others in different cancer models, it was shown that CAFs in the bone marrow of MM patients can originate from mesenchymal stem cells, from endothelial cells and hematopoietic stem cells. Furthermore, *in vitro* experiments showed that CAFs sustain MM cell proliferation and render them more resistant to apoptosis; *in vivo* experiments demonstrated that co-injection of MM cells with CAFs favors tumor take and growth. We also demonstrated that the CAFs-MM cell interaction involves CXCL12/CXCR4 pathway and several integrins confirming the importance of contact between tumor and stromal cells. CAFs were also found to sustain MM disease indirectly by stimulating the angiogenic process by recruitment of endothelial cells in the tumor [76].

Proteomic profiling by Slany’s group identified different proteins in fibroblast-like cells of non-neoplastic control patients, MGUS patients and MM patients. Proteins involved in ECM remodeling (e.g., laminin α4, integrin α5β5, nidogen-2, prolyl-4-hydroxylase) were progressively upregulated in MGUS and MM, indicating an important role of fibroblasts in MM progression [77].

In MM, a small subpopulation of CD138^−^ cells has been identified within the CD38^+^ MM cell population. CD138^−^ cells were less sensitive to treatment (including bortezomib and melphalan) compared to CD138^+^ cells and could possibly contribute to the high incidence of relapse of MM patients. Co-culture experiments of MM cells with stromal cell lines or primary MSC cultures increased the number of the CD138^−^ cell fraction, implying the involvement of the bone marrow microenvironment in relapsed MM patients [78,79]. Although little is known about the effects of currently used myeloma drugs on CAFs, a paper by Celegato *et al.* showed bortezomib was able to downregulate CD49d and CD44 expression factors that mediate the adhesion of human Hodgkin lymphoma cells to fibroblasts, indicating effects of myeloma treatment on CAFs [80]. Together, all these data demonstrated that CAFs in the bone marrow niche have an important role sustaining the progression of MM disease.

## 10. Hypoxic Niche in Multiple Myeloma

Hypoxia results from an imbalance between the oxygen (O_2_) supply and the O_2_ consumption in cells or tissues. Decreased O_2_ levels are observed in several pathological conditions including cancer. Hypoxia not only increases gene amplification and genomic instability in the cancer cells, but also induces expression of pro-angiogenic factors (e.g., VEGF), and ABC transporters, and promotes the invasive capacity of the tumor cells [81,82,83]. Up to 60% of the advanced solid tumors exhibit hypoxic regions throughout the tumor mass, which are associated with resistance to chemotherapy and radiation. Although hypoxia plays a crucial role in normal BM hematopoiesis, the role in hematological malignancies is barely investigated. In MM patients, increased levels of hypoxia-inducible factor (HIF)-1α and HIF-2α, key factors involved in hypoxia responses, were determined in bone marrow (BM) biopsies [84]. In addition, hypoxia was also investigated in the bone marrow of naïve and 5T33MM mice. Data clearly showed a significant increase in the exogenous hypoxia marker pimonidazole and the endogenous marker HIF-1α in 5T33MM diseased mice, indicating that MM cells reside in a more hypoxic bone marrow microenvironment [81]. Several strategies to target hypoxia are currently under investigation including the hypoxia-activated prodrug TH-302. *In vivo* treatment of 5T33MM mice with TH-302 induced apoptosis of MM cells in the bone marrow microenvironment and decreased paraprotein secretion [85]. Storti and colleagues also showed that selective HIF-1α inhibition reduced tumor burden *in vivo* and strongly decreased angiogenesis and the development of osteolytic bone lesions [86]. Hypoxia not only affects tumor growth, but also activates epithelial to mesenchymal transition-like features in MM cells and metastasis [87]. Together, these data indicate an emerging role of hypoxia in tumor growth and progression, making it an interesting target for cancer therapy including MM.

## 11. Role of CAFs and Hypoxia in Tumor Progression

Little is known about how hypoxia regulates CAF recruitment and activation. A loss in caveolin-1 (Cav-1), a marker highly expressed in terminally differentiated stromal cells, was described as an important driver of HIF-1α expression and was associated with an activated CAF phenotype. Briefly, a crosstalk between HIF-1α and Cav-1 induces autophagy/mitophagy in the tumor stroma, and consequently provides cancer cells with essential amino acids and nucleotides to drive tumor growth and metastasis. Cancer cells themselves are protected against apoptosis due to the upregulation of the autophagy inhibitor TIGAR [88]. Furthermore, it has been demonstrated that hypoxia increases oxidative stress, which in turn can induce myofibroblast differentiation through accumulation of HIF-1α and increased expression of CXCL12. Oxidative stress was able to enhance the migration of fibroblasts and to increase tumor growth [89]. Chaivarina and colleagues stably-expressed HIF-1α in fibroblasts and found a 3-fold increase in tumor volume, indicating a relation between CAFs, hypoxia and tumor progression [90]. Higgens and colleagues further described hypoxia as a regulator of the ECM and TGF-β signaling pathway, both important factors in the CAF generation [91]. A recent publication demonstrated that arginase-2 (Arg2) was especially expressed in CAFs derived from pancreatic ductal carcinoma tissue. L-arginine serves as a substrate for Arg2 and nitric oxide synthase (NOS). Hypoxia was able to induce Arg2 expression in CAFs and these cells were able to create a more immunosuppressive microenvironment [92].

**Figure 3:**
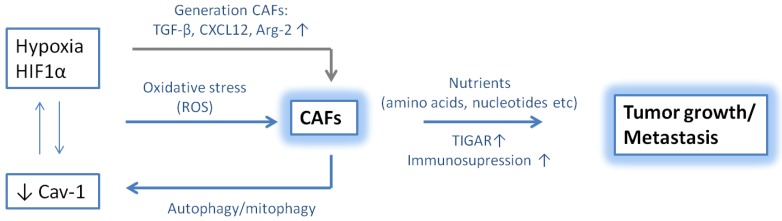
The relation between hypoxia and cancer associated fibroblasts.

A recent publication by Kim and colleagues investigated different fibroblast-specific conditional knockout mice using a FSP-cre strain. In contrast to previous data, they observed that ablation of HIF-1α in stromal fibroblasts was able to increase tumor growth and perfusion [93]. Although HIF-1α is generally regarded as a tumor-promoting factor in endothelial cells, epithelial cells and myeloid cells, these data indicate that distinct processes can occur depending on the cell type (Figure 3). 

## 12. Conclusions

CAFs are a heterogeneous subpopulation of fibroblasts, extensively studies in solid carcinomas. They become activated by distinct features including growth factors, mechanical stress and hypoxic conditions, which characterizes the growing tumors. Furthermore, CAFs influences tumor cell proliferation through physical interaction, cytokine secretion and interactions with other cell types including endothelial cells and immune cells. Although the role of CAFs in hematological malignancies is unclear, recent data indicate that CAFs are important mediators of tumor progression in MM. MM is a plasma cell cancer characterized by a hypoxic bone marrow niche, which may favor the fibroblast activation. It is evident that the contribution of CAFs to tumor progression and metastasis cannot be neglected and needs to be further clarified.
